# Exopolysaccharide Isolated from *Lactobacillus plantarum* L-14 Has Anti-Inflammatory Effects via the Toll-Like Receptor 4 Pathway in LPS-Induced RAW 264.7 Cells

**DOI:** 10.3390/ijms21239283

**Published:** 2020-12-05

**Authors:** Mijin Kwon, Jaehoon Lee, Sangkyu Park, Oh-Hee Kwon, Jeongmin Seo, Sangho Roh

**Affiliations:** 1 Cellular Reprogramming and Embryo Biotechnology Laboratory, Dental Research Institute, Seoul National University School of Dentistry, Seoul 08826, Korea; rnjsalwls23@hanmail.net (M.K.); good0039@hanmail.net (S.P.); 2 Biomedical Research Institute, NeoRegen Biotech Co., Ltd., Gyeonggi-do 16614, Korea; jaylee6322@gmail.com (J.L.); ohee@neoregenbio.com (O.-H.K.)

**Keywords:** postbiotics, exopolysaccharide, lipopolysaccharide, Toll-like receptor 4, inflammation, oxidative stress

## Abstract

Inflammation is a biological response of the immune system to defend the body from negative stimulation. However, the excessive inflammatory response can damage host tissues and pose serious threats. Exopolysaccharide (EPS), one of the postbiotics, is secreted from lactic acid bacteria. Although many studies have described the beneficial effects of EPS, such as its anti-inflammatory and anti-oxidant effects, its underlying mechanisms have remained to be poorly understood. Thus, we identified that EPS obtained from *Lactobacillus plantarum* L-14 was a homogeneous polysaccharide primarily comprised of glucose. To examine these anti-inflammatory effects, an inflammatory response was induced by lipopolysaccharide (LPS) administration to mouse macrophage RAW 264.7 cells that were pretreated with EPS. The anti-inflammatory effects of EPS were identified by analyzing the changes within inflammatory markers at the molecular level. We demonstrate here that EPS suppressed proinflammatory mediators, such as cyclooxygenase-2, interleukin-6, tumor necrosis factor-α, and interleukin-1β, and downregulated the expression of an inducible nitric oxide synthase known to lead to oxidative stress. It was also confirmed that EPS had anti-inflammatory effects by blocking the interaction of LPS with Toll-like receptor 4 (TLR4), as demonstrated by using the known TLR4 inhibitor TAK-242. In addition, we found that EPS itself could suppress the expression of TLR4. Consequently, our data suggest that EPS can be a potential target for the development of natural product-derived medicine for treating inflammatory diseases related to TLR4.

## 1. Introduction

Inflammation has been identified as an essential defense reaction to protect the body from various harmful stimuli, such as infection, toxic compounds, or injury [1]. However, upon aggravation of the inflammatory response, the host may undergo tissue damage and potentially contract a chronic inflammatory disorder such as rheumatoid arthritis, psoriasis, lupus erythematosus, and asthma [2]. Inflammatory bowel disease affects more than 1.4 million people in the USA, with an average annual direct medical cost of 6.3 billion USD [3]. Clearly, systemic regulation of inflammation plays a significant role for disease progression within individuals and strongly impacts public health [4]. Inflammatory reactions have been determined to be related to various diseases, and a number of anti-inflammatory drugs have been developed thus far. For example, aspirin—a non-steroidal anti-inflammatory—is one of the most commonly prescribed drugs for suppressing inflammation and pain. Adalimumab, a TNF-α monoclonal antibody drug, has been effective in treating inflammatory diseases such as rheumatoid arthritis, psoriatic arthritis, ankylosing spondylitis, and Crohn’s disease. Although a number of drugs for regulating inflammatory reactions have been studied and developed, a multitude of issues remain in terms of their usage, such as non-targeting effects and high prices [5].

Toll-like receptors are determined to be major players in the defense system of the host. In particular, Toll-like receptor 4 (TLR4) is known as a critical driver of the innate immune response to bacterial infections, and its dysregulation can contribute to a variety of diseases, such as: asthma, cardiovascular disease, metabolic syndrome, autoimmune disorders, and even schizophrenia [6]. Activation of TLR4 stimulates the mitogen-activated protein kinase (MAPK) and nuclear factor kappa-light-chain-enhancer of activated B cells (NF-κB) pathway; consequently, it facilitates the gene expression of inflammatory mediators like cyclooxygenase-2 (COX-2), interleukin-6 (IL-6), tumor necrosis factor-α (TNF-α), and interleukin-1β (IL-1β) [7]. Lipopolysaccharide (LPS) as one of the components in the outer membrane of gram-negative bacteria has a pathogen-associated molecular pattern that is recognized by TLR4. When gram-negative bacterial cells are lysed and LPS binds to TLR4 on host cells, the inflammatory response is triggered upon activation of the innate immune system. The resulting inflammatory reaction can then cause fever, diarrhea, cardiac dysfunctions, and, in some cases, even death [8,9]. Therefore, TLR4 could be a therapeutic target for treating immune diseases.

According to the World Health Organization, probiotics are defined as “live microorganisms which give a health benefit to the host when administered in adequate amounts” [10]. The probiotics market was expected to grow 37% globally from 2016 to 2020 due to such beneficial effects as improvement of intestinal health and prevention of insulin resistance [11,12]. However, recent studies have indicated that periodic intake of probiotics could lead to unexpected adverse effects. For example, administration of probiotics could result in infections, undesired inflammatory response, and gene transfer from probiotics to natural host microbiota [13]. Postbiotics, also known as “simply metabolites” or “cell-free supernatants,” are identified as bioactive compounds secreted by live lactic acid bacteria (LAB) [14]. According to Kareem et al., postbiotics can be used as a substitute for probiotics since the former can exhibit probiotic effects without the risk of transferring antibiotic resistance genes to the host [15]. The studies for postbiotic effects have shown that their compounds directly interact with the host and can have positive reactions [16]. Postbiotics can improve intestinal barrier function, protect the intestine from *Escherichia coli* (*E. coli*) pathogenesis, and even induce apoptosis, specifically on cancer cells [17,18]. Additionally, Malagón-Rojas et al. demonstrated that postbiotics can be recommended for children younger than 5 years of age due to rare risk of LAB-related infections in infants, such as pneumonia and meningitis [19]. Since postbiotics are stable over a wide range of pH and temperatures and can be separated into individual components, they are suitable for in vitro and in vivo studies and are easy to commercialize [20]. Exopolysaccharide (EPS), one of the postbiotics secreted by LAB, can interact with host cells by acting as ligands and protect the host by aggregating with pathogens in the intestine [21]. In addition, it has been reported that EPS exhibits protective effects from oxidative stress as well as anti-cancer benefits [22,23]. Interestingly, a recent study has shown that EPS can prevent viral infections by modulating an antiviral immune response within intestinal epithelial cells [24]. However, the mechanisms involved in the beneficial effects of EPS have remained to be unknown. We treated RAW 264.7 cells with EPS in an attempt to identify which mechanism(s) are mediated during the regulation of the inflammatory response by EPS from *Lactobacillus plantarum* (*L. plantarum*) L-14.

EPS isolated from L-14 growth media was identified as a polysaccharide primarily composed of glucose. To confirm any potential anti-inflammatory effects, EPS was pretreated in RAW 264.7 cells, and stimulation of the inflammatory response was then induced by LPS. The cytokines and proteins related to the inflammatory pathway were downregulated in the EPS pretreatment group. To examine whether EPS inhibits the interaction between LPS and TLR4, the TLR4 pathway was analyzed using the known TLR4 inhibitor TAK-242. The activation of TLR4 and myeloid differentiation factor (MyD88) by LPS is then suppressed by EPS. Furthermore, we could identify that EPS itself suppressed TLR4 expression without LPS stimulation.

## 2. Results

### 2.1. Co-Culture with L-14 Inhibited the Induction of Inflammation by LPS in RAW 264.7 Cells

**Figure 1:**
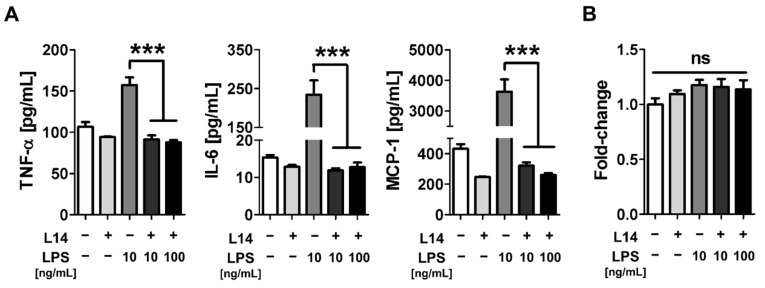
To determine whether L-14 could suppress the inflammatory response, RAW 264.7 cells were co-cultured with L-14 for 6 h and then treated 1 μg/mL LPS for 6 h. (**A**) The production of cytokines stimulated by LPS was quantified via ELISA. Co-culture with L-14 significantly inhibited the release of tumor necrosis factor-α (TNF-α), interleukin-6 (IL-6), and monocyte chemoattractant protein-1 into the media. (**B**) Viability was not affected by L-14 in RAW 264.7 cells, which suggests that L-14 exhibited anti-inflammatory effects by directly interacting with immune cells. *** *p* < 0.001 versus the LPS-treated group; ns, not significant.

To determine whether co-culture with L-14 suppressed the inflammatory response via interaction with immune cells, the cytokines induced by LPS in mouse macrophage RAW 264.7 cells were quantified via enzyme-linked immunosorbent assay (ELISA). The cells seeded in a 12-well plate were co-cultured with L-14 for 6 h, and the proinflammatory markers IL-6, TNF-α, and monocyte chemoattractant protein-1 (MCP-1) were induced by LPS. As a result, the expression levels of IL-6, TNF-α, and MCP-1 were increased by LPS, but not by L-14 (Figure 1A). Surprisingly, the release of inflammatory cytokines was significantly decreased to control the levels in cells that had been previously co-cultured with L-14. To confirm whether these results were caused by L-14 affecting cell proliferation, the viability of the RAW 264.7 cells cultured under the same culture conditions was quantified by WST-8 cell viability assay, which showed that cell viability was unaffected by L-14 or LPS (Figure 1B). These results suggested that the metabolite secreted from L-14 exhibited anti-inflammatory effects by directly interacting with immune cells.

### 2.2. EPS Isolated from L-14 Was a Homogeneous Polysaccharide Primarily Composed of Glucose

**Figure 2:**
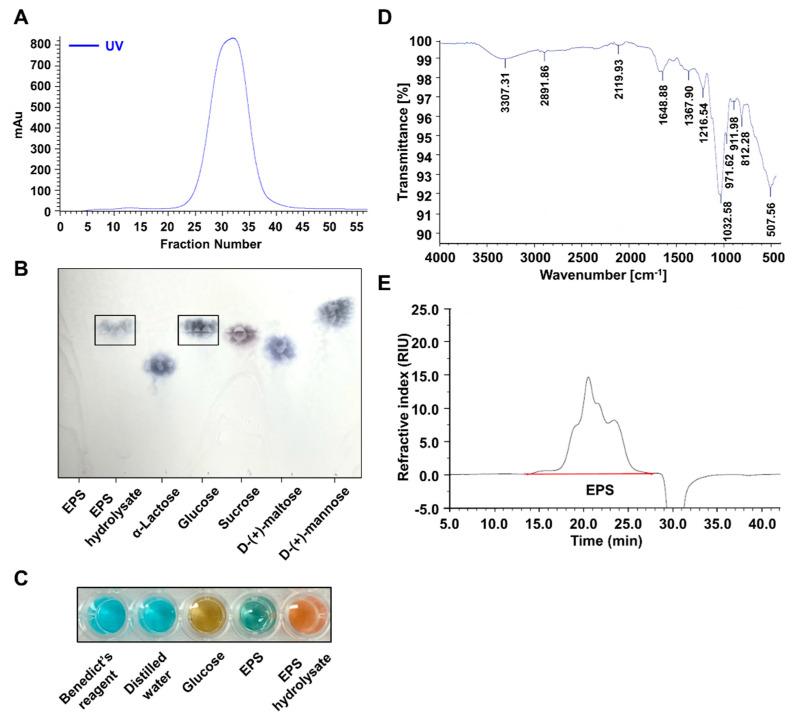
Exopolysaccharide (EPS) isolated from L-14 was polysaccharide mainly composed of glucose. (**A**) EPS isolated from L-14 culture media was identified as a homogeneous polysaccharide by Fast protein liquid chromatography size exclusion chromatography. (**B**) Thin layer chromatography analysis showed that the monosaccharide component of EPS was primarily composed of glucose. (**C**) Benedict’s test confirmed that EPS was a polysaccharide that mainly consists of glucose. (**D**) The result of Fourier-transform infrared spectroscopy showed that EPS had the characteristic peaks of polysaccharides. (**E**) The weight average molecular weight (Mw) of EPS was determined as 7.57 × 10^4^ Da by Gel Permeation Chromatography.

EPS was purified from culture media via ethanol precipitation. Isolated EPS was analyzed by fast protein liquid chromatography (FPLC) size exclusion chromatography, resulting in a single symmetrical peak, indicating that EPS was a homogeneous polysaccharide (Figure 2A). The monosaccharide components of EPS were determined by Thin layer chromatography (TLC) (Figure 2B). EPS hydrolysate was expressed at the same point with the glucose used as the standard, so the results indicated that EPS was mainly composed of glucose. We conducted Benedict’s test to confirm that EPS is primarily composed of glucose. EPS hydrolysate changed the color of the reagent into orange-red (Figure 2C). Additionally, the color of the reagent was changed into green by EPS. The characteristic structure of EPS was analyzed using Fourier-transform infrared spectroscopy (FTIR). The result of FTIR showed a complex pattern of peaks from 3500 to 500 cm^−1^ (Figure 2D). The peaks indicated the characteristic group of glucose such as the presence of O-H groups at 3307.31 cm^−1^, a weak C-H stretching peak of methyl groups at 2935.1 cm^−1^, and C=O stretching at 1648.88 cm^−1^ [25]. Additionally, the strongest absorption band, 1032.58 cm^−1^, was assigned to C–O bond and O-H bond, identifying the presence of polysaccharides [26]. The bands at 911.98 and 812.28 cm^−1^ corresponded to the side-group of carbohydrates [27]. Thus, the results showed that EPS had the absorption peaks of polysaccharides mainly composed of glucose. To measure the molecular weight of EPS, EPS was analyzed through Gel Permeation Chromatography (GPC) with pullulan standards (Figure 2E). When the molecular weight is calculated by the chromatography data system, EPS has a number average molecular weight (Mn) of 1.84 × 10^4^ Da, a weight average molecular weight (Mw) of 7.57 × 10^4^ Da, a size average molecular weight (Mz) of 3.74 × 10^5^ Da, and a polydispersity index (PDI) (Mw/Mn) of 4.12. These results indicated that EPS was a homogeneous polysaccharide mainly composed of glucose.

### 2.3. EPS Isolated from L-14 Alleviated Morphological Changes Induced by LPS within Mouse Macrophages

**Figure 3:**
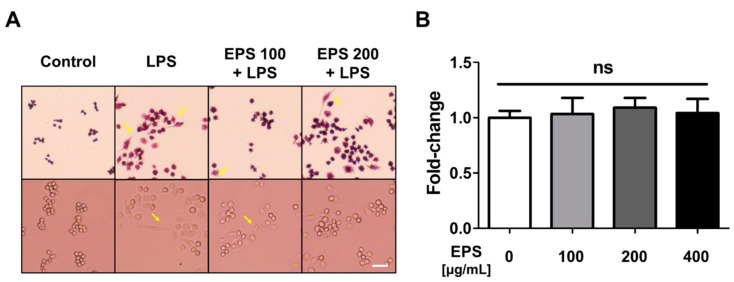
The purified EPS could alleviate the morphological changes induced by LPS in RAW 264.7 cells. (**A**) Cells pretreated with EPS were cultured with fresh media containing LPS and stained with crystal violet. EPS pretreatment inhibited the LPS-induced morphological changes, as indicated by the yellow arrow. (**B**) EPS treatment did not have any effect upon cell viability after 1 day. Scale bar = 50 μm; ns, not significant.

To confirm whether EPS alleviated the morphological changes induced by LPS, cells were pretreated with EPS for 6 h, and then, the morphological changes were stimulated by LPS for 18 h. Crystal violet staining showed that EPS treatment could alleviate the alteration of cell morphology in LPS-treated cells (Figure 3A). Furthermore, when RAW 264.7 cells were treated with a higher concentration of EPS for 1 day, cell viability remained unaffected (Figure 3B). These results indicated that EPS inhibited LPS-induced morphological changes in mouse macrophages without impacting cell viability.

### 2.4. LPS-Induced Inflammatory Response Was Inhibited by EPS Pretreatment

**Figure 4:**
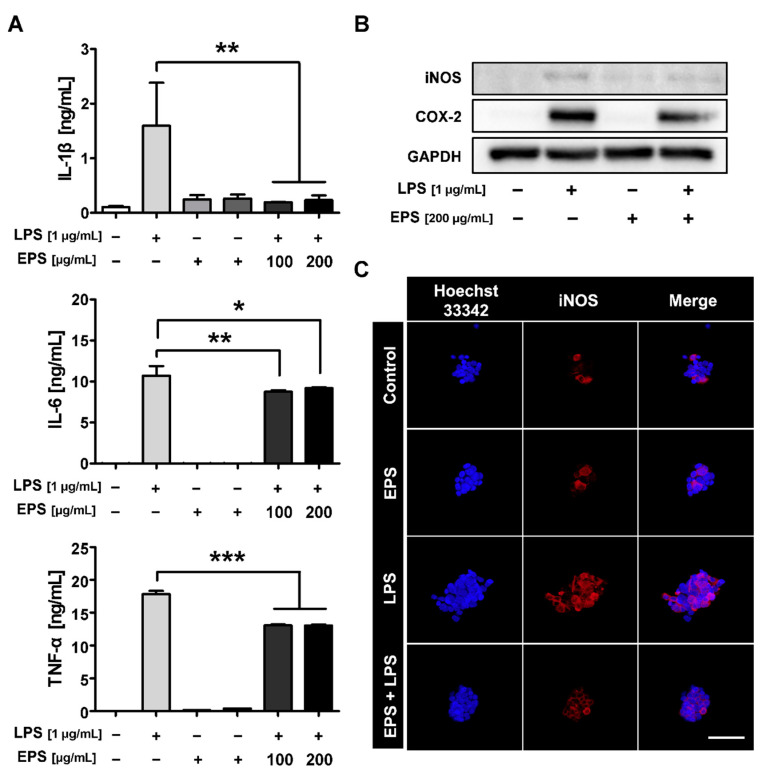
EPS inhibited the LPS-induced inflammatory response in RAW 264.7 cells. (**A**) Cells were pretreated with EPS, and proinflammatory cytokines were quantified using ELISA; EPS attenuated the expression levels of IL-6, TNF-α, and interleukin-1β (IL-1β). (**B**) EPS suppressed the expression of cyclooxygenase-2 (COX-2) and inducible nitric oxide synthase (iNOS) proteins induced by LPS in RAW 264.7 cells. (**C**) The expression of iNOS stimulated by LPS was inhibited in EPS-pretreated RAW 264.7 cells as shown by immunofluorescence (IF) assay. * *p* < 0.05, ** *p* < 0.01, and *** *p* < 0.001 versus the LPS-treated group. Scale bar = 50 μm.

To confirm whether the isolated EPS inhibited the inflammatory response resulting from LPS stimulation, we quantified the proinflammatory cytokines produced by RAW 264.7 cells precultured with EPS. EPS pretreatment attenuated IL-6, TNF-α, and IL-1β levels in culture media, and in particular, IL-1β was decreased similar to the expression levels of the control (Figure 4A). Furthermore, the expression of COX-2 and inducible nitric oxide synthase (iNOS), known as major mediators of inflammation, was analyzed through Western blot. While the expressions of COX-2 and iNOS proteins were increased following LPS treatment, these expression levels were suppressed in EPS-pretreated RAW 264.7 cells (Figure 4B). Consistent with these results, immunofluorescence (IF) assay also confirmed that the expression of LPS-induced iNOS was decreased in EPS-pretreated RAW 264.7 cells (Figure 4C). Taken together, these results indicate that EPS purified from L-14 exhibited suppression upon the LPS-stimulated inflammatory response.

### 2.5. EPS Inhibited Nuclear Translocation of NF-κB Induced by LPS

**Figure 5:**
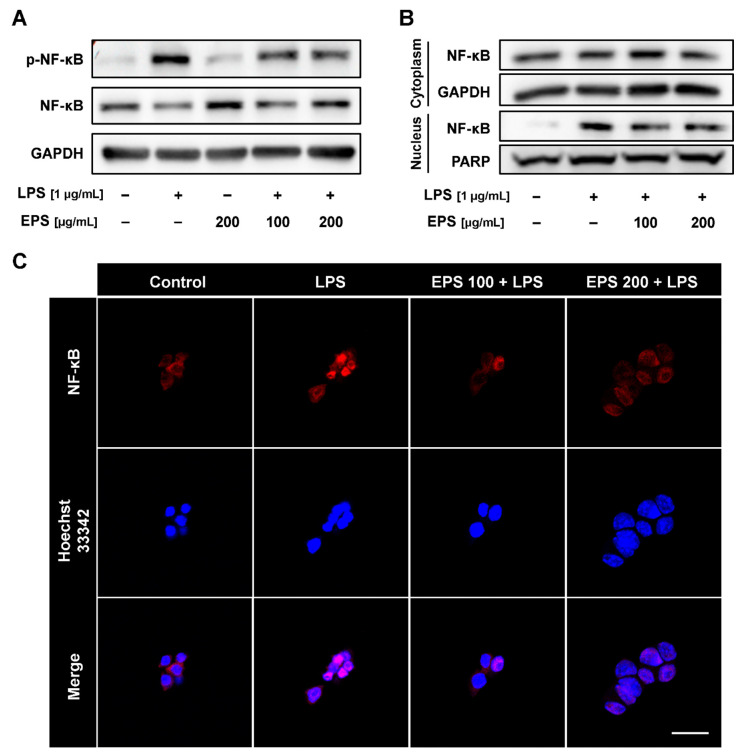
EPS has inhibited the phosphorylation and nuclear translocation of nuclear factor kappa-light-chain-enhancer of activated B cells (NF-κB) induced by LPS. (**A**) Phosphorylation of NF-κB was suppressed by EPS in LPS-treated cells as shown via Western blot. (**B**) EPS decreased the translocation of NF-κB into the nucleus following LPS treatment. (**C**) IF assay also showed that EPS inhibited LPS-induced nuclear translocation of NF-κB at all tested concentrations. Scale bar = 20 μm.

To examine whether EPS inhibited phosphorylation and translocation to the nucleus of NF-κB following induction by LPS, the inflammatory response was stimulated by LPS in RAW 264.7 cells pretreated with EPS followed by an analysis of the expression level of NF-κB and localization of its phosphorylated form. The p-NF-κB/NF-κB ratio was then decreased by EPS pretreatment (Figure 5A). EPS itself did not promote the phosphorylation of NF-κB. As shown in Figure 5B, EPS suppressed LPS-induced nuclear translocation of NF-κB at all concentrations. Consistently, while translocation of NF-κB into the nucleus was induced by LPS, this was reduced by pretreatment of EPS in RAW 264.7 cells (Figure 5C).

### 2.6. EPS-Repressed Inflammatory Response via Regulation of MAPK and Nuclear Factor E2-Related Factor 2 (NRF2)/Heme Oxygenase-1 (HO-1) Pathways in RAW 264.7 Cells

**Figure 6:**
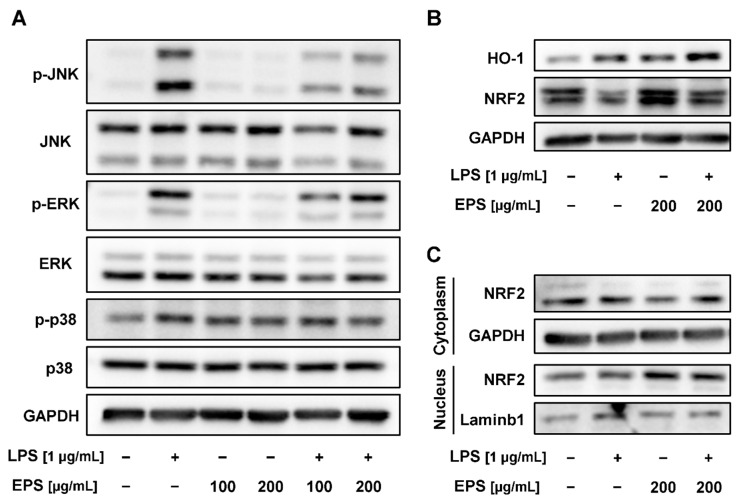
EPS regulated mitogen-activated protein kinase (MAPK) and Nuclear factor E2-related factor 2 (NRF2)/Heme oxygenase-1 (HO-1) pathways known as major pathways mediating the inflammatory response. (**A**) To determine that EPS could inhibit the MAPK pathway in LPS-induced RAW 264.7 cells, the phosphorylation in MAPK family proteins was analyzed through Western blot. The result showed that EPS inhibited the phosphorylation of JNK and ERK induced by LPS, although that of p38 was less inhibited. (**B**) Expression of both HO-1 and NFR2 was upregulated with or without the presence of LPS. (**C**) EPS stimulated the translocation of NRF2 into the nucleus.

The MAPK and NRF2/HO-1 pathways are identified as major regulators of the inflammatory response in mouse macrophages. To determine whether EPS has any effects on the MAPK pathway, phosphorylation of MAPK family proteins (JNK, ERK, and p38) was analyzed. EPS significantly inhibited the LPS-induced phosphorylation of JNK and ERK even at 100 μg/mL of EPS (Figure 6A). Phosphorylation of p38 resulting from LPS was suppressed in the cells treated with EPS at a concentration of 200 μg/mL. To confirm that the anti-inflammatory effects of EPS were mediated through the NRF2/HO-1 pathway, the protein expression levels of known anti-oxidant markers were identified. The expression levels of both HO-1 and NFR2 were then increased with or without LPS (Figure 6B). Consistent with the previous results, the translocation of NRF2 into the nucleus was also increased following EPS exposure (Figure 6C). In summary, EPS inhibited the phosphorylation of MAPK family proteins and enhanced the expression of NRF2/HO-1 in LPS-induced RAW 264.7 cells.

### 2.7. EPS Inhibits the Inflammatory Response by Suppressing the Interaction between LPS and TLR4

**Figure 7:**
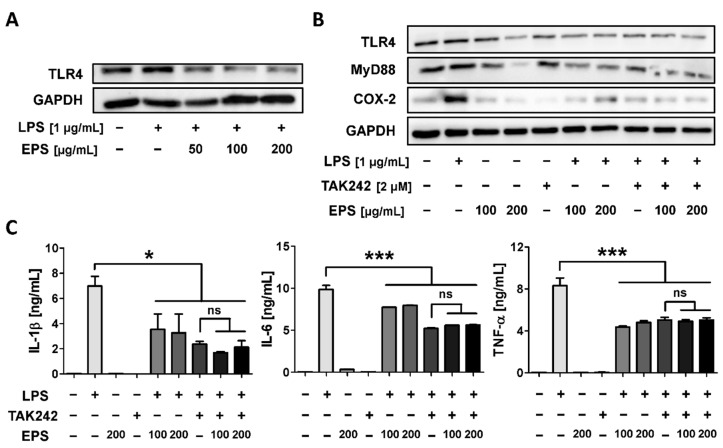
EPS inhibited LPS-induced inflammatory response through Toll-like receptor 4 (TLR4) pathway in RAW 264.7 cells. (**A**) EPS inhibited the expression of TLR4 induced by LPS. (**B**) The expression levels of TLR4, Myeloid differentiation factor (MyD88), and COX-2 in the EPS-pretreated groups were analyzed using TAK-242; expression levels of TLR4 and MyD88 were inhibited by EPS in LPS-treated groups similar to the TAK-242-treated group; COX-2 was inhibited by TAK-242 and EPS to a similar level. (**C**) EPS and TAK-242 suppressed the expression of proinflammatory cytokines induced by LPS. However, there was no synergetic effect observed when EPS and TAK-242 were simultaneously administered to RAW 264.7 cells. * *p* < 0.05 and *** *p* < 0.001 versus the LPS-treated group; ns, not significant.

To examine whether EPS suppressed the inflammatory response via TLR4, the expression level of TLR4 was analyzed following EPS and LPS treatment of RAW 264.7 cells. As a result, TLR4 upregulated by LPS was reduced by EPS at all concentrations (Figure 7A). In addition, the manner in which EPS interacted with TLR4 was confirmed using TAK-242, which has been determined to block the TLR4 pathway. Interestingly, when RAW 264.7 cells were treated with EPS only, the protein expression of TLR4 was more repressed than that of the no treatment control group (Figure 7B). EPS also inhibited the expression levels of TLR4 and MyD88 in LPS-induced groups, similar to that observed in the TAK-242-treated group. The expression of COX-2 was inhibited by TAK-242 and was inhibited by EPS in the same way. Consistently, EPS significantly reduced the expression of the secreted cytokines IL-1β, IL-6, and TNF-α in the media as much as TAK-242 downregulated them (Figure 7C). The results suggested that EPS exhibited anti-inflammatory effects through TLR4 in LPS-treated RAW 264.7 cells.

## 3. Discussion

Inflammatory reactions are considered a common biological response to pathogens, which occurs in all tissues and organs [28]. Inflammation is deemed essential for the elimination of harmful stimuli and damaged cells and initiation of tissue repair involving immune cells and molecular mediators. However, an uncontrolled or excessive inflammatory response can contribute to chronic inflammatory diseases and, in some instances, even death [29,30]. Thus, a number of attempts have been performed to develop therapeutics to treat diseases caused by unregulated inflammation [31]. Recently, it has been reported that postbiotic compounds are capable of regulating the immune responses of various animal models [32]. For example, EPS produced by *Lactobacillus delbrueckii* inhibited the inflammatory response induced by *E. coli* via the IκB pathway in porcine intestinal epithelial cells [33]. However, the receptor interacting with EPS and related mechanisms remain poorly understood.

When LPS binds to TLR4, the interaction can induce an inflammatory response and oxidative stress by NF-κB and MAPK signaling pathway in the host cells [7]. TLR4 signaling is divided into two pathways, that is, the MyD88-dependent pathway and TIR-domain-containing adapter-inducing interferon-β (TRIF)-dependent pathway, of which the mutual interaction generates a complicated inflammation response [34]. TNF-α expression induced by LPS is regulated through both MyD88 and TRIF pathways, whereas NF-κB is primarily activated through a MyD88-dependent pathway [35]. Our results have shown that EPS has inhibited the expression of TNF-α and NF-κB following their activation by LPS (Figure 4A and Figure 5). In some recent research, it was shown that LPS accumulated in the brain neurons of patients with Alzheimer’s disease and inhibited the efficient readout of neuronal genetic information for the homeostasis of brain cell function by contributing to inflammatory degeneration in human neuronal-glial cells [36]. Rathinam et al. showed that TLR4-deficient mice did not develop an immune response following injections of LPS at high concentrations, which only suggests that the inflammatory reaction caused by LPS may be completely regulated through TLR4 pathway [37]. EPS obtained from LAB could be divided into homopolysaccharide (HoPS) composed of the same monosaccharides and heteropolysaccharide consists of various monosaccharides [16]. In particular, HoPS is polymerized from monosaccharides such as glucose or fructose by glycosyl hydrolase activity and released out from the cell [38]. HoPS is produced through either intracellular synthesis, which undergoes several metabolic pathways or extracellular synthesis, which is released immediately after polymerization. Therefore, HoPS has different ligands depending on the methods it is made. These complex structural properties contribute to the biological function, characteristics, or beneficial effects of EPS. EPS isolated L-14 was identified as HoPS mainly composed of glucose (Figure 2B–D). The PDI of EPS was calculated as 4.12, and EPS changed the color of the Benedict solution to green, indicating that EPS contained a small amount of glucose. (Figure 2C,E). These results confirmed that EPS is a broad polydisperse polysaccharide composed of monosaccharides of glucose. Interestingly, our results showed that EPS isolated from L-14 significantly inhibited the expression of proinflammatory markers—including IL-6, TNF-α, IL-1β, and COX-2—induced by LPS, indicating that EPS suppressed the TLR4 pathway (Figure 4 and Figure 7A). In addition, our findings have confirmed that EPS inhibited the activation of TLR4 by LPS, as demonstrated by TAK-242 treatment (Figure 7B,C). When cells were treated with both EPS and TAK-242, TLR4 and proinflammatory mediators were not more repressed than when cells were treated with either agent independently. These results suggested the possibility that EPS inhibits the inflammatory response via a mechanism similar to that of TAK-242. TLR4 directly binds to not only LPS but also products of gram-positive bacteria such as EPS and lipoteichoic acid. Recently, it was reported that the EPS produced by *Bifidobacterium animalis* was able to interact with TLR4 of intestinal epithelial cells [39]. Nevertheless, it is necessary to elaborate in detail what structure of EPS from L-14 has and whether the ligand of EPS directly binds to TLR4 like TAK-242. Anti-cytokine therapy has been widely used to treat immune diseases in the last decade [40]. Such approved drugs include inhibitors of TNF-α (e.g., etanercept and adalimumab), IL-6 (e.g., tocilizumab), and IL-1β (e.g., canakinumab) signaling for immune diseases such as rheumatoid arthritis and Crohn’s disease [41]. Surprisingly, proinflammatory cytokines are also found to be highly expressed in major depression disorder (MDD) patients [42]; in particular, TNF-α stimulates the development of MDD by changing the function and structure of the brain [43]. In randomized trials for MDD treatment, depressive symptoms were shown to decrease by up to 43.8% after taking anti-TNF-α agents (i.e., etanercept, adalimumab, and golimumab) [44]. Furthermore, IL-1β is known as the major cytokine contributing to cardiac ischemia reperfusion injury due to activation through the inflammasome. Cytokines present in the myocardium, including IL-1β, TNF-α, and IL-6, have even resulted in life-threatening ventricular arrhythmias through modulation of potassium and calcium channels [45]. Administration of anti-IL-1β monoclonal antibody reduced the relative risk of major adverse cardiovascular events by up to 25%, in addition to the 31% decline in cardiovascular and overall mortality in patients with a history of myocardial infarction [46]. However, injection of therapeutic monoclonal antibody has commonly resulted in adverse reactions, including infections and reactions at the injection site [47]. Our experiment showed that EPS can effectively reduce the secretion of proinflammatory cytokines without cytotoxicity in macrophages (Figure 3B and Figure 4). The inflammatory response of macrophages can promote the development of the innate and adaptive immune response by complex interactions with other immune cells, including natural killer cells and dendritic cells [48]. EPS could be used to inhibit inflammatory reactions in macrophages and further treat diseases caused by unregulated inflammatory reactions through oral intake.

EPS has been determined to upregulate the expression of NRF2/HO-1 and consequently decreased iNOS, a known producer of nitric oxide (NO), one of reactive oxygen species (ROS), in LPS-induced macrophages (Figure 4B,C and Figure 6B,C). Oxidative stress often results in the damage of DNA and proteins, mitochondrial dysfunction, and apoptosis, which can lead to age-related diseases such as chronic inflammatory diseases and cancer [49]. Additionally, excessive ROS causing oxidative stress in cells are considered as risks and enhancer factors for chronic inflammatory diseases [50]. The activation of the anti-oxidant molecules NRF2/HO-1 exhibited a clinical benefit through anti-oxidant effects in animal models with rheumatoid arthritis [51]. Recently, it has been reported that symptoms of neurodegenerative diseases deteriorated when deficiency of NRF2 increased neuroinflammation and oxidative stress in mice [52]. Additionally, it was reported that oxidative stress can be provoked by activating the MAPK pathway, since the members of the ROS and MAPK families can positively regulate each other [53]. For example, hydrogen peroxide treatment increases the expression of genes that can activate the MAPK pathway; likewise, oxidative stress was suppressed via treatment with the MAPK inhibitors PD98059 (ERK inhibitor) and SB203580 (p38 inhibitor) [54]. Taken together, the regulation of the NRF2/HO-1 and MAPK pathways has been determined to suppress ROS production, which leads to the reduction of the oxidative stress response. Although the inhibitory activities of EPS were not observed in a dose-dependent manner, phosphorylation of JNK, ERK, and p38 was decreased by EPS (Figure 6A). Consistently, the expression of iNOS has reportedly decreased at the protein level (Figure 4B,C). Interestingly, even though p38 was phosphorylated by EPS itself, EPS did not significantly affect the expression of iNOS (Figure 4B,C and Figure 6A). Although the NO level was not quantified and it was not determined how iNOS and the MAPK pathway regulated each other, it appears indisputable that EPS retains the ability to reduce oxidative stress. Therefore, EPS could be a potential substance to manage ROS levels, and, consequently, prevent and treat the diseases caused by oxidative stress.

TLR4 has been applied as a target for immunopharmacological control of infection from pathogenic bacteria and even viruses [55]. Especially, viral infection can lead to a cytokine storm, which develops via overexpressed proinflammatory mediators, making the immune cells unable to prevent cytokine production [56]. A recent study has confirmed that the coronavirus causing COVID-19 stimulates a TLR4-mediated inflammatory response similar to the pathogenic process of bacterial sepsis [57]. Furthermore, it has been reported that the innate immune response during Ebola virus infection begins when the viral glycoprotein binds to TLR4; subsequently, NF-κB and MAPK signaling pathways are activated [58]. Shirey et al. confirmed the possibility for TLR4 antagonists (e.g., eritoran) to be used as novel therapeutics for the influenza virus-induced cytokine storm [59]. Liu Shen Wan, a known traditional medicine of China, even inhibited the proliferation of the virus as well as expression of cytokines by suppressing TLR4 [60]. Interestingly, our results have shown that EPS itself downregulated TLR4 and MyD88 compared with the control group (Figure 7B); this suggests that it can act as an inhibitor of TLR4. Thus, EPS could be used to prevent and treat the diseases caused by uncontrolled inflammatory response associated with virus infections. It has also been shown that TLR4 is involved in the maintenance of host homeostasis [61]. Knockdown of TLR4 ameliorates insulin resistances and glucose tolerance, suggesting that TLR4 is a key therapeutic target for metabolic disorders [62]. Withaferin A decreases the gene expression of TLR4 and COX-2, which protects high-fat diet-induced mice against metabolic disorders such as glucose tolerance, insulin resistance, and oxidative stress [63]. Expression of COX-2 in the EPS treatment group was more downregulated than that of the control group by suppressing TLR4, regardless of the presence of LPS (Figure 7B). It has already been reported that curcumin, known as a TLR4 inhibitor, can be a candidate for the treatment of metabolic syndrome and type II diabetes mellitus through a clinical trial test [64]. Therefore, EPS could potentially be used to treat metabolic diseases as well as diseases resulting from bacterial and viral infections. However, it is essential to confirm the effect EPS has on normal cells and whether EPS can exhibit anti-inflammatory effect in vivo.

**Figure 8:**
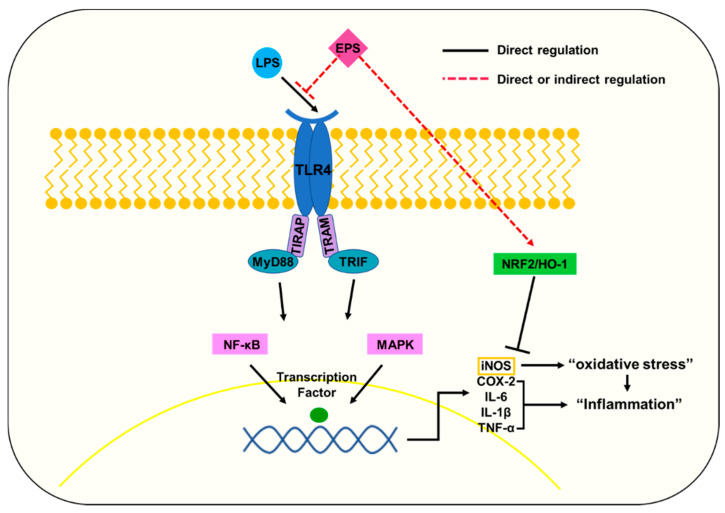
When LPS binds to TLR4 in the cell membrane, the resulting complex can lead to the activation of adaptor proteins including MyD88 and TIR-domain-containing adapter-inducing interferon-β (TRIF). NF-κB is phosphorylated and translocated into the nucleus by adaptor proteins. MyD88 and TRIF phosphorylate members of the MAPK pathway, and the MAPK family protein translocates transcription factors related to the inflammatory response into the nucleus. These translocated factors upregulate the expression of proinflammatory cytokines and COX-2. However, EPS could inhibit the LPS-induced inflammatory response by downregulating TLR4. EPS also repressed the expression of iNOS, which led to oxidative stress by upregulating the NRF2/HO-1 pathways. Nevertheless, more research is required to elucidate how EPS regulates the expression of TLR4.

In conclusion, EPS isolated from *L. plantarum* L-14 was polysaccharide mainly composed of glucose and inhibited proinflammatory mediators such as those of the NF-κB and MAPK pathways by suppressing TLR4 and MyD88 signaling (Figure 8). This suggests that EPS could be an attractive candidate for natural product-derived medicine in regulating acute or chronic inflammatory reactions. It remains unclear how EPS itself decreases the expression of TLR4 or whether EPS regulates them directly or indirectly. Follow-up study is required to elaborate the clear interaction between EPS and TLR4.

## 4. Materials and Methods

### 4.1. Material

Antibodies were purchased from the following sources: phospho-NF-κB, NF-κB, phospho-ERK, ERK, phospho-p38, p38, phospho-JNK, JNK, and COX-2 from Cell Signaling Technology (Danvers, MA, USA), HO-1 from Abcam (Cambridge, UK), NRF2 and TLR4 from Cusabio (Wuhan, China), iNOS from Invitrogen (Carlsbad, CA, USA), MyD88 from Novus Biologicals (Centennial, CO, USA), and GAPDH from BioLegend (San Diego, CA, USA).

### 4.2. L-14 Culture and Exopolysaccharide Purification

The L-14 strain (KTCT13497BP), which was purchased from NeoRegen Biotech (Suwon, Gyeonggi-do, Korea), was cultured at 30 °C for 18 h in Man, Rogosa and Sharpe (MRS; Hardy Diagnostics, Santa Maria, CA, USA) broth, which contained 2% dextrose, 1% peptic digest of animal tissue, 1% beef extract, 0.5% yeast extract, 0.5% sodium acetate, 0.2% disodium phosphate, 0.2% ammonium citrate, 0.1% polysorbate 80, 0.01% magnesium sulfate, and 0.005% manganese sulfate. EPS was purified using the ethanol precipitation method as previously described [65]. In summary, L-14 culture media was separated via centrifugation at 10,000× *g* for 20 min. Media supernatant was then isolated, and trichloroacetic acid was added to denature proteins in the L-14-cultured media at 37 °C for 1 h. Denatured proteins were isolated from media by centrifugation at 10,000× *g* for 20 min, followed by mixing with absolute ethanol. The separated precipitates were dialyzed with the distilled water (DW) at 4 °C for 24~48 h to fully remove the components of media and other substances. The dialyzed solution was then lyophilized to obtain EPS, which was resuspended in DW for subsequent experiments and stored −80 °C.

### 4.3. FPLC

To identify EPS as a homogeneous polysaccharide, EPS (30 mg/mL) was purified by size exclusion chromatography on a HiLoad^®^ 16/600 Superdex 200 pg column (GE Healthcare, Chicago, IL, USA) with phosphate-buffered saline (PBS), and it was analyzed via the ÄKTA FPLC system (GE Healthcare).

### 4.4. TLC and Benedict’s Test

To identify the monosaccharide composition of EPS through TLC, 10 mg of EPS was hydrolyzed with 1 mL sulfuric acid (2 N) at 100 °C for 4 h. The residual sulfuric acid was neutralized with the enough BaCO3 for 12 h. After EPS hydrolysate was adjusted to PH 7, it was lyophilized for the analysis.

EPS hydrolysate was treated on TLC Silica gel (Merck, Darmstadt, Germany) and migrated with the buffer composed with n-butanol:methanol:25% ammonia solution:DW (5:4:2:1). To visualize the composition of EPS, the gel was soaked with aniline-diphenylamine reagent and baked in the oven at 110 °C for 5 min.

To conduct Benedict’s test, EPS and EPS hydrolysate were mixed with the same quantify of Benedict’s reagent (BIOZOA Biological Supply, Seoul, Korea) and then heated in boiling water.

### 4.5. FTIR and GPC

To confirm the structural characteristics of EPS, EPS was analyzed using TENSOR27 FTIR (Bruker, Billerica, MA, USA) in the absorption range from 4000 to 500 cm^−1^ by National Center for Inter-University Research Facilities of Seoul National University.

To determine the molecular weight of EPS, GPC analysis was performed using Dionex HPLC Ultimate3000 RI System (Thermo Scientific, Waltham, MA, USA) with 120 Å, 500 Å, and 1000 Å columns (Waters, Milford, MA, USA) at 40 °C by National Instrumentation Center for Environmental Management of Seoul National University. The data of the experiment was calibrated with pullulan and processed with the chromatography data system (Chromeleon 6.8 Extention-pak). EPS was eluted using Sodium azide 0.1 M in water and operated at a flow rate of 1 mL/min.

### 4.6. Cell Culture

The mouse macrophage cell line RAW 264.7 was obtained from the American Type Culture Collection. The cells were cultured in Dulbecco’s modified Eagle’s media (WELGENE, Daegu, Korea), which contained 10% fetal bovine serum (HyClone, Logan, UT, USA) and 1% penicillin/streptomycin (Gibco, Grand Island, NY, USA). The cells were cultured at 37 °C in an incubator with a humidified atmosphere of 5% CO_2_.

### 4.7. Cell Viability Assay

RAW 264.7 cells were seeded at a density of 2.0 × 10^3^ cells per well in 96-well plates. A day after, media was replaced with L-14 media and cultured for another 6 h. The media containing L-14 was then replaced with fresh media containing LPS to induce inflammatory response at the indicated concentrations for 6 h. Cell viability was confirmed using Quanti-Max WST-8 cell viability kit (BIOMAX, Seoul, Korea). To determine the effect of EPS on cell viability, the seeded cells were incubated in media containing EPS at various concentrations for 1 day. Cell viability was verified by the same kit.

### 4.8. ELISA

RAW 264.7 cells were seeded into 12-well plates at a density of 2.0 × 10^5^ cells per well. The media was then replaced with L-14-inoculated media at a concentration of 1.0 × 10^6^ CFU/mL and maintained for 6 h. The media was then removed, and cells were washed thoroughly three times with DMEM. To induce inflammation, the washed cells were cultured in media containing LPS (1 μg/mL) and maintained for 6 h. The culture media obtained from each well was centrifuged at 10,000× *g* for 3 min, and the supernatants were collected. Cytokines were quantified by ELISA MAX™ Deluxe Set (BioLegend) according to the manufacturer’s recommendations. To examine whether pretreatment of EPS decreased the induction of cytokines by LPS, RAW 264.7 cells were seeded into 12-well plates for 24 h. The cells were pretreated with EPS for 6 h, and then the cultured media was replaced with fresh media containing LPS for 18 h to induce an inflammatory response. The cytokines in the culture media were quantified as described above.

### 4.9. Crystal Violet Staining

RAW 264.7 cells were seeded into 12-well plates and incubated for 1 day. To examine whether EPS pretreatment has affected the morphology of cells and inhibited the morphological changes induced by LPS, cells were treated with EPS for 6 h, and the cultured media was replaced with the fresh media containing LPS (1 μg/mL) for 18 h. Then, the cells were washed with PBS and stained with crystal violet solution (Sigma-Aldrich, Saint Louis, MO, USA). The morphological changes were determined using an EVOS CL Core microscope (Life Technologies, Carlsbad, CA, USA) at 100× magnification.

### 4.10. Western Blot

The proteins were isolated from RAW 264.7 cells treated with LPS using Cell Culture Lysis 1× Reagent (Promega, Fitchburg, WI, USA) with a protease inhibitor cocktail and phosphatase inhibitor cocktail. The cytoplasmic and nuclear proteins were obtained using ExKine™ Nuclear and Cytoplasmic Protein Extraction Kit (Abbkine, Wuhan, China) according to the manufacturer’s instructions. The total protein concentration was quantified by Pierce™ BCA Protein Assay Kit (Thermo Scientific, Waltham, MA, USA). The denatured protein was then separated by 12% sodium dodecyl sulfate-polyacrylamide gel electrophoresis and transferred to a polyvinylidene difluoride membrane. After blocking for 1 h at room temperature (RT), the membrane was incubated in skim milk containing the proper primary antibodies (1:1000) overnight at 4 °C. The membrane was washed using tris-buffered saline with 0.1% Tween 20 (Sigma) and was further incubated in skim milk containing secondary antibodies (1:2000) for 1 h at RT. The protein signals were detected using the ECL Western Blot Substrate (Daeil Lab Service, Seoul, Korea).

### 4.11. IF Assay

RAW 264.7 cells were seeded at a density of 1.0 × 10^6^ cells per well in 6-well plates and were incubated overnight. EPS-pretreated cells were cultured in fresh media containing LPS and harvested by scraping. After fixation with 4% paraformaldehyde and permeabilization by 0.1% Triton X-100 (Sigma), the cells were blocked with 3% bovine serum albumin (BSA, Bovogen, East Keilor, Australia) for 1 h; then, they were incubated with PE-conjugated iNOS antibody. The washed cells were mounted with ProLong™ Glass Antifade Mountant with NucBlue™ Stain (Invitrogen).

To visualize the translocation of NF-κB, RAW 264.7 cells were pretreated with EPS for 2 h, and the inflammatory response was stimulated by LPS (1 μg/mL) for 1 h. The isolated cells were prepared via the same method. After blocking for 1 h, cells were incubated with NF-κB antibody (1:200) for 18 h at 4 °C. The cells were then washed thoroughly and incubated in 3% BSA with Alexa Fluor 488-conjugated secondary antibodies for 30 min at RT. Cells were mounted using the same stain. All slides were analyzed using LSM 800 confocal laser scanning microscope 293 (Carl Zeiss, Oberkochen, Germany).

### 4.12. Statistics

In this study, all data were obtained from three independent experiments and presented with mean ± standard deviation (SD). Statistical analysis was determined using unpaired ANOVA, and significance was defined as * *p* < 0.05, ** *p* < 0.01, and *** *p* < 0.001.

## Abbreviations

BSA	Bovine serum albumin
COX-2	Cyclooxygenase-2
DW	Distilled water
*E. coli*	*Escherichia coli*
ELISA	Enzyme-linked immunosorbent assay
EPS	Exopolysaccharide
FPLC	Fast protein liquid chromatography
FTIR	Fourier-transform infrared spectroscopy
GPC	Gel permeation chromatography
HO-1	Heme oxygenase-1
HoPS	Homopolysaccharide
IF	Immunofluorescence
IL-1β	Interleukin-1β
IL-6	Interleukin-6
iNOS	Inducible nitric oxide synthase
*L. plantarum*	*Lactobacillus plantarum*
LAB	Lactic acid bacteria
LPS	Lipopolysaccharide
MAPK	Mitogen-activated protein kinase
MCP-1	Monocyte chemoattractant protein-1
MDD	Major depression disorder
MyD88	Myeloid differentiation factor
NF-κB	Nuclear factor kappa-light-chain-enhancer of activated B cells
NO	Nitric oxide
NRF2	Nuclear factor E2-related factor 2
PBS	Phosphate-buffered saline
PDI	Polydispersity index
ROS	Reactive oxygen species
RT	Room temperature
SD	Standard deviation
TLC	Thin layer chromatography
TLR4	Toll-like receptor 4
TNF-α	Tumor necrosis factor-α
TRIF	TIR-domain-containing adapter-inducing interferon-β

## References

[1] Ferrero-Miliani L., Nielsen O.H., Andersen P.S., Girardin S.E. (2007). Chronic inflammation: Importance of NOD2 and NALP3 in interleukin-1beta generation. Clin. Exp. Immunol..

[2] Gaestel M., Kotlyarov A., Kracht M. (2009). Targeting innate immunity protein kinase signalling in inflammation. Nat. Rev. Drug Discov..

[3] Huang H., Fang M., Jostins L., Mirkov M.U., Boucher G., Anderson C.A., Andersen V., Cleynen I., Cortes A., Crins F. (2017). Fine-mapping inflammatory bowel disease loci to single-variant resolution. Nature.

[4] Chen L., Deng H., Cui H., Fang J., Zuo Z., Deng J., Li Y., Wang X., Zhao L. (2018). Inflammatory responses and inflammation-associated diseases in organs. Oncotarget.

[5] de Anda-Jáuregui G., Guo K., McGregor B.A., Hur J. (2018). Exploration of the anti-inflammatory drug space through network pharmacology: Applications for drug repurposing. Front. Physiol..

[6] Lucas K., Maes M. (2013). Role of the toll like receptor (TLR) radical cycle in chronic inflammation: Possible treatments targeting the TLR4 pathway. Mol. Neurobiol..

[7] Li P.-Y., Liang Y.-C., Sheu M.-J., Huang S.-S., Chao C.-Y., Kuo Y.-H., Huang G.-J. (2018). Alpinumisoflavone attenuates lipopolysaccharide-induced acute lung injury by regulating the effects of anti-oxidation and anti-inflammation both in vitro and in vivo. RSC Adv..

[8] Yücel G., Zhao Z., El-Battrawy I., Lan H., Lang S., Li X., Buljubasic F., Zimmermann W.-H., Cyganek L., Utikal J. (2017). Lipopolysaccharides induced inflammatory responses and electrophysiological dysfunctions in human-induced pluripotent stem cell derived cardiomyocytes. Sci. Rep..

[9] Li Y., Huang X., Huang S., He H., Lei T., Saaoud F., Yu X.-Q., Melnick A., Kumar A., Papasian C.J. (2017). Central role of myeloid MCPIP1 in protecting against LPS-induced inflammation and lung injury. Signal Transduct. Target. Ther..

[10] (2001). Health and Nutritional Properties of Probiotics in Food Including Powder Milk with Live Lactic Acid Bacteria. http://pc.ilele.hk/public/pdf/20190225/bd3689dfc2fd663bb36def1b672ce0a4.pdf.

[11] Kamiński M., Łoniewski I., Marlicz W. (2019). Global internet data on the interest in antibiotics and probiotics generated by Google Trends. Antibiotics.

[12] Natural Products Insider. https://www.naturalproductsinsider.com/digestive-health/new-market-profile-probiotics-consumption.

[13] Sotoudegan F., Daniali M., Hassani S., Nikfar S., Abdollahi M. (2019). Reappraisal of probiotics’ safety in human. Food Chem. Toxicol..

[14] Izuddin W.I., Loh T.C., Samsudin A.A., Foo H.L. (2018). In vitro study of postbiotics from *Lactobacillus plantarum* RG14 on rumen fermentation and microbial population. Rev. Bras. Zootecn..

[15] Kareem K.Y., Loh T.C., Foo H.L., Akit H., Samsudin A.A. (2016). Effects of dietary postbiotic and inulin on growth performance, IGF1 and GHR mRNA expression, faecal microbiota and volatile fatty acids in broilers. BMC Vet. Res..

[16] Zhou Y., Cui Y., Qu X. (2019). Exopolysaccharides of lactic acid bacteria: Structure, bioactivity and associations: A review. Carbohydr. Polym..

[17] Chuah L.-O., Foo H.L., Loh T.C., Alitheen N.B.M., Yeap S.K., Mutalib N.E.A., Rahim R.A., Yusoff K. (2019). Postbiotic metabolites produced by *Lactobacillus plantarum* strains exert selective cytotoxicity effects on cancer cells. BMC Complement. Altern. Med..

[18] Gao J., Li Y., Wan Y., Hu T., Liu L., Yang S., Gong Z., Zeng Q., Wei Y., Yang W. (2019). A novel postbiotic from *Lactobacillus rhamnosus GG* with a beneficial effect on intestinal barrier function. Front. Microbiol..

[19] Malagón-Rojas J.N., Mantziari A., Salminen S., Szajewska H. (2020). Postbiotics for preventing and treating common infectious diseases in children: A systematic review. Nutrients.

[20] Barros C.P., Guimarães J.T., Esmerino E.A., Duarte M.C.K., Silva M.C., Silva R., Ferreira B.M., Sant’Ana A.S., Freitas M.Q., Cruz A.G. (2020). Paraprobiotics and postbiotics: Concepts and potential applications in dairy products. Curr. Opin. Food Sci..

[21] Castro-Bravo N., Wells J.M., Margolles A., Ruas-Madiedo P. (2018). Interactions of surface exopolysaccharides from *Bifidobacterium* and *Lactobacillus* within the intestinal environment. Front. Microbiol..

[22] Li J.-Y., Jin M.-M., Meng J., Gao S.-M., Lu R.-R. (2013). Exopolysaccharide from *Lactobacillus planterum* LP6: Antioxidation and the effect on oxidative stress. Carbohydr. Polym..

[23] Saadat Y.R., Khosroushahi A.Y., Gargari B.P. (2019). A comprehensive review of anticancer, immunomodulatory and health beneficial effects of the lactic acid bacteria exopolysaccharides. Carbohydr. Polym..

[24] Kanmani P., Albarracin L., Kobayashi H., Iida H., Komatsu R., Kober A.H., Ikeda-Ohtsubo W., Suda Y., Aso H., Makino S. (2018). Exopolysaccharides from *Lactobacillus delbrueckii* OLL1073R-1 modulate innate antiviral immune response in porcine intestinal epithelial cells. Mol. Immunol..

[25] Pană A.-M., Rusnac L.-M., Bandur G., Silion M., Deleanu C., Bălan M. (2011). Novel D-glucose and D-mannose based oligomers: Synthesis and characterization. E-Polymers.

[26] Nataraj S., Schomäcker R., Kraume M., Mishra I., Drews A. (2008). Analyses of polysaccharide fouling mechanisms during crossflow membrane filtration. J. Membr. Sci..

[27] Gieroba B., Krysa M., Wojtowicz K., Wiater A., Pleszczyńska M., Tomczyk M., Sroka-Bartnicka A. (2020). The FT-IR and Raman spectroscopies as tools for biofilm characterization created by cariogenic streptococci. Int. J. Mol. Sci..

[28] Medzhitov R. (2010). Inflammation 2010: New adventures of an old flame. Cell.

[29] Zhou Y., Hong Y., Huang H. (2016). Triptolide attenuates inflammatory response in membranous glomerulo-nephritis rat via downregulation of NF-κB signaling pathway. Kidney Blood Press. Res..

[30] Czaja A.J. (2014). Hepatic inflammation and progressive liver fibrosis in chronic liver disease. World J. Gastroenterol..

[31] Serhan C.N. (2017). Treating inflammation and infection in the 21st century: New hints from decoding resolution mediators and mechanisms. FASEB J..

[32] Aguilar-Toalá J., Garcia-Varela R., Garcia H., Mata-Haro V., González-Córdova A., Vallejo-Cordoba B., Hernández-Mendoza A. (2018). Postbiotics: An evolving term within the functional foods field. Trends Food Sci. Tech..

[33] Wachi S., Kanmani P., Tomosada Y., Kobayashi H., Yuri T., Egusa S., Shimazu T., Suda Y., Aso H., Sugawara M. (2014). *Lactobacillus delbrueckii* TUA 4408 L and its extracellular polysaccharides attenuate enterotoxigenic *E scherichia coli*-induced inflammatory response in porcine intestinal epitheliocytes via T oll-like receptor-2 and 4. Mol. Nutr. Food Res..

[34] Guo J., Friedman S.L. (2010). Toll-like receptor 4 signaling in liver injury and hepatic fibrogenesis. Fibrogenesis Tissue Repair.

[35] Sakai J., Cammarota E., Wright J.A., Cicuta P., Gottschalk R.A., Li N., Fraser I.D., Bryant C.E. (2017). Lipopolysaccharide-induced NF-κB nuclear translocation is primarily dependent on MyD88, but TNFα expression requires TRIF and MyD88. Sci. Rep..

[36] Zhao Y., Cong L., Lukiw W.J. (2017). Lipopolysaccharide (LPS) accumulates in neocortical neurons of Alzheimer’s disease (AD) brain and impairs transcription in human neuronal-glial primary co-cultures. Front. Aging. Neurosci..

[37] Rathinam V.A., Zhao Y., Shao F. (2019). Innate immunity to intracellular LPS. Nat. Immunol..

[38] Gupta P., Diwan B. (2017). Bacterial exopolysaccharide mediated heavy metal removal: A review on biosynthesis, mechanism and remediation strategies. Biotechnol. Rep..

[39] Castro-Bravo N., Margolles A., Wells J.M., Ruas-Madiedo P. (2019). Exopolysaccharides synthesized by *Bifidobacterium animalis* subsp. lactis interact with TLR4 in intestinal epithelial cells. Anaerobe.

[40] Rider P., Carmi Y., Cohen I. (2016). Biologics for targeting inflammatory cytokines, clinical uses, and limitations. Int. J. Cell. Biol..

[41] Goralski K.B., Ladda M.A., McNeil J.O. (2018). Drug-cytokine interactions. Drug Interactions in Infectious Diseases: Mechanisms and Models of Drug Interactions.

[42] Dantzer R., O’Connor J.C., Freund G.G., Johnson R.W., Kelley K.W. (2008). From inflammation to sickness and depression: When the immune system subjugates the brain. Nat. Rev. Neurosci..

[43] Ma K., Zhang H., Baloch Z. (2016). Pathogenetic and therapeutic applications of tumor necrosis factor-α (TNF-α) in major depressive disorder: A systematic review. Int. J. Mol. Sci..

[44] Wu C.-Y., Chang Y.-T., Juan C.-K., Shen J.-L., Lin Y.-P., Shieh J.-J., Liu H.-N., Chen Y.-J. (2016). Depression and insomnia in patients with psoriasis and psoriatic arthritis taking tumor necrosis factor antagonists. Medicine.

[45] Lazzerini P.E., Laghi-Pasini F., Boutjdir M., Capecchi P.L. (2019). Cardioimmunology of arrhythmias: The role of autoimmune and inflammatory cardiac channelopathies. Nat. Rev. Immunol..

[46] Ridker P.M., MacFadyen J.G., Everett B.M., Libby P., Thuren T., Glynn R.J., Kastelein J., Koenig W., Genest J., Lorenzatti A. (2018). Relationship of C-reactive protein reduction to cardiovascular event reduction following treatment with canakinumab: A secondary analysis from the CANTOS randomised controlled trial. Lancet.

[47] Baldo B.A. (2016). Other Approved Therapeutic Monoclonal Antibodies.

[48] Navegantes K.C., de Souza Gomes R., Pereira P.A.T., Czaikoski P.G., Azevedo C.H.M., Monteiro M.C. (2017). Immune modulation of some autoimmune diseases: The critical role of macrophages and neutrophils in the innate and adaptive immunity. J. Transl. Med..

[49] Ruiz S., Pergola P.E., Zager R.A., Vaziri N.D. (2013). Targeting the transcription factor Nrf2 to ameliorate oxidative stress and inflammation in chronic kidney disease. Kidney Int..

[50] Schieber M., Chandel N.S. (2014). ROS function in redox signaling and oxidative stress. Curr. Biol..

[51] Cuadrado A., Manda G., Hassan A., Alcaraz M.J., Barbas C., Daiber A., Ghezzi P., León R., López M.G., Oliva B. (2018). Transcription factor NRF2 as a therapeutic target for chronic diseases: A systems medicine approach. Pharmacol. Rev..

[52] Brandes M.S., Gray N.E. (2020). NRF2 as a therapeutic target in neurodegenerative diseases. ASN. Neuro..

[53] Zhang J., Wang X., Vikash V., Ye Q., Wu D., Liu Y., Dong W. (2016). ROS and ROS-mediated cellular signaling. Oxid. Med. Cell. Longev..

[54] Bohush A., Niewiadomska G., Filipek A. (2018). Role of mitogen activated protein kinase signaling in Parkinson’s disease. Int. J. Mol. Sci..

[55] Mukherjee S., Karmakar S., Babu S.P.S. (2016). TLR2 and TLR4 mediated host immune responses in major infectious diseases: A review. Braz. J. Infect. Dis..

[56] Sang Y., Miller L.C., Blecha F. (2015). Macrophage polarization in virus-host interactions. J. Clin. Cell. Immunol..

[57] Sohn K.M., Lee S.-G., Kim H.J., Cheon S., Jeong H., Lee J., Kim I.S., Silwal P., Kim Y.J., Park C. (2020). COVID-19 patients upregulate toll-like receptor 4-mediated inflammatory signaling that mimics bacterial sepsis. J. Korean Med. Sci..

[58] Lai C.-Y., Strange D.P., Wong T.A.S., Lehrer A.T., Verma S. (2017). Ebola virus glycoprotein induces an innate immune response in vivo via TLR4. Front. Microbiol..

[59] Shirey K.A., Lai W., Scott A.J., Lipsky M., Mistry P., Pletneva L.M., Karp C.L., McAlees J., Gioannini T.L., Weiss J. (2013). The TLR4 antagonist Eritoran protects mice from lethal influenza infection. Nature.

[60] Ma Q., Huang W., Zhao J., Yang Z. (2020). Liu Shen Wan inhibits influenza a virus and excessive virus-induced inflammatory response via suppression of TLR4/NF-κB signaling pathway in vitro and in vivo. J. Ethnopharmacol..

[61] Lu Y., Li X., Liu S., Zhang Y., Zhang D. (2018). Toll-like Receptors and Inflammatory Bowel Disease. Front. Immunol..

[62] Zhao Y., Li G., Li Y., Wang Y., Liu Z. (2017). Knockdown of TLR4 in the arcuate nucleus improves obesity related metabolic disorders. Sci. Rep..

[63] Bakar M.H.A., Azmi M.N., Shariff K.A., Tan J.S. (2019). Withaferin A protects against high-fat diet–induced obesity via attenuation of oxidative stress, inflammation, and insulin resistance. Appl. Biochem. Biotechnol..

[64] Ain Q.U., Batool M., Choi S. (2020). TLR4-targeting therapeutics: Structural basis and computer-aided drug discovery approaches. Molecules.

[65] Bajpai V.K., Majumder R., Rather I.A., Kim K. (2016). Extraction, isolation and purification of exopolysaccharide from lactic acid bacteria using ethanol precipitation method. Bangladesh J. Pharmacol..

